# Midwives’ experiences with aortic compression for postpartum hemorrhage: A qualitative study

**DOI:** 10.18332/ejm/172880

**Published:** 2023-11-23

**Authors:** Sonja Kalsvik, Mirjam Lukasse, Enid L. Myhre

**Affiliations:** 1 Centre for Women’s, Family and Child Health, Faculty of Health Sciences, University of South-Eastern Norway, Kongsberg, Norway

**Keywords:** guidelines, maternal mortality, postpartum haemorrhage, aortic compression, first-line response

## Abstract

**INTRODUCTION:**

The aim of this study was to examine midwives’ firsthand experience with aortic compression during postpartum hemorrhage. Severe postpartum hemorrhage is a critical complication during childbirth and the leading cause of maternal morbidity and mortality. Active management of the third stage of labor, combined with standard treatment, has reduced the incidence. However, these measures occasionally fall short, and there is a global need for easy, effective alternative methods. Aortic compression, though not widely recognized, is employed intermittently and lacks substantial scientific backing.

**METHODS:**

This qualitative study comprised interviews with midwives from various healthcare settings across Norway. Over a two-month period in 2022, we conducted seven individual semi-structured interviews. Interview transcripts were thematically analyzed using Braun and Clarke’s six-step process.

**RESULTS:**

Four prominent themes emerged from the analysis, reflecting midwives’ experiences with aortic compression in managing postpartum hemorrhage (PPH). In their experiences with aortic compression, midwives uncovered its dual qualities of being both easy and effective. Their utilization of the technique was experience-based only, shaped by personal experience rather than formal training. Nevertheless, aortic compression was perceived as the first-line response to suspected postpartum hemorrhage, preventing escalation, and offering a clearer view of the situation to facilitate timely treatment. Most significantly, midwives recognized aortic compression as a crucial intervention that reduces blood loss and improves health.

**CONCLUSIONS:**

Postpartum hemorrhage is a feared situation in the delivery room. The participants consider that aortic compression may affect maternal health and mortality. However, further research is necessary.

## INTRODUCTION

Postpartum hemorrhage (PPH) refers to excessive bleeding after childbirth, commonly defined as more than 500 mL^1^. Severe PPH (>1000 mL) occurs in approximately 5% of all deliveries^1,2^, is the leading cause of maternal mortality worldwide, and is likely to cause morbidities (e.g. anemia, infections, lactation problems and/or fatigue)^3^. More than 90% of all maternal deaths occur in low- and middle-income countries and 65% occur in Africa^4^. PPH risk factors include multiple pregnancy, previous cesarean section, pre-existing anemia, prolonged labor, maternal age, induction of labor, and pre-eclampsia^5^.

Treatment of PPH commonly consists of medications, massaging the uterus, catheterizing the bladder, crede and/or manual emptying of the uterus^4^. These interventions do not always stop the bleeding, resulting in the woman’s condition rapidly deteriorating. In these cases, uterine balloon tamponade, uterine compression sutures, resuscitative endovascular balloon occlusion of the aorta (REBOA), and embolization, may be tried^6^. REBOA and embolization aim to occlude the aorta and blood flow to the uterus^6-9^; these are surgical procedures that require trained staff, a surgical unit, and access to X-ray technology. As a last resort, hysterectomy could be performed^4^.

An alternative method, which can be used in settings with limited facilities, is external aortic compression. External aortic compression is performed by pressing a closed fist down, just above and to the left of the umbilicus, with the intend to compress the aorta externally. The aortic pulsation should be felt on the fist and the pressure should continue until the pulsation below the fist ceases^10,11^. The method is mentioned in older obstetric literature^12,13^ and used in humanitarian aid work, but there is little knowledge about its use and prevalence. The World Health Organization (WHO)^4^ describes external aortic compression as part of PPH interventions but recognizes the need for further research.

Aortic compression can also be used in highly specialized care settings in combination with other methods, to limit bleeding while preparing for more advanced procedures. The method is used in Norway, but its prevalence and how it is taught to or experienced by midwives is unknown. This study sought to explore the experiences of midwives who use aortic compression in their work.

## METHODS

We conducted a qualitative study exploring midwives’ experiences with aortic compression during PPH. Data were collected by semi-structured interviews, over a two-month period.

### Sampling and participants

Initial recruitment aimed at recruiting all midwives from one hospital where aortic compression is regularly used. Information about the study was posted on the ward via leaflets, presented at handovers, and in a general email to all midwives^14^. This recruited too few participants, so information was posted on a closed Facebook group, inviting midwives with relevant experience to participate in the study. The ‘snowball’ method was also used, asking participants for the contact information of midwives who had used aortic compression. The only inclusion criteria were that the midwives had used aortic compression during PPH. Of the seven midwives participating in the study, three came from the initially targeted hospital ward, three through the Facebook post, and one through the snowball sampling.

### Ethics

Approval from the Norwegian Centre for Research Data was granted and the study was conducted in accordance with the World Medical Association Declaration of Helsinki Ethical Principles for Medical Research Involving Human Subjects^15^. Participants were given verbal and written information about the purpose of the study. They were informed that participation was voluntary, and they could withdraw at any time, without explanation. Written consent was obtained from all participants. Participants were ensured anonymity, and that the data would be stored safely and deleted upon completion of the analysis.

### Data collection and analysis

Five of the interviews were conducted digitally through Zoom, and two were face-to-face. The first author conducted all interviews, using an interview guide with open-ended questions (Supplementary file). Two questions were added to the interview guide after the first interviews, including ‘Where did you learn the method?’ and ‘Are there any patients where you think aortic compression will not be suited?’. Clarification and follow-up questions were asked when needed. The interviews were conducted in January and February 2022 and lasted from 10 to 41 minutes (mean 21 minutes). We sought to achieve data saturation, the point at which new information and themes ceased to emerge from the interviews. We conducted a total of seven interviews, and it became evident during the analysis that no new substantial insights were provided in the later interviews. The interviews were audio recorded and transcribed verbatim. Braun and Clarke’s thematic analysis method was used to analyze the data^16,17^.

Thematic analysis followed Braun and Clarke’s^17^ six-step processes. First, we familiarized ourselves with the data. The first author (SK) transcribed the interviews consecutively, and SK and the last author (ELM) re-read all transcripts and noted initial ideas. In the second step, initial codes were generated. All codes were grouped into potential subthemes and main themes; each theme was then related to the original codes and the entire data set (steps 4 and 5). The codes and themes were identified and organized using NVivo (version 12; QSR International). In step five, we examined the overall story of the analyses, seeking themes relevant to aortic compression. In the final step, we agreed upon clear definitions and names for the themes and subthemes, ensuring that each also fits into the overarching narrative. We reached a consensus on the themes after discussing them with all three authors during the final stage of the analysis. Following a semantic approach, thematic analysis was only carried out on data relevant to how the midwives experienced aortic compression. The data, coding, subthemes and main themes were considered carefully, to ensure a proper analysis.

## RESULTS

Of the seven participating midwives, three had experience from one specific hospital where aortic compression is routinely practiced. The remaining four midwives, who did not work in the targeted hospital, brought diverse experiences to the study. Three of them had experience from humanitarian aid work in addition to their Norwegian practice. One participant contributed valuable insights based on her experience in Sweden. Participants’ level of experience in aortic compression varied widely, with some having used the technique a few times, while others reported more extensive exposure.

Four main themes emerged from the analyses concerning midwives’ experience with aortic compression: ‘easy and effective’, ‘experience-based only’, ‘first-line response’, and ‘reduces blood loss and improves health’ (Table 1). The four themes collectively illuminate the multifaceted aspects of midwives’ encounters with aortic compression during PPH.

**Table 1:** Main themes and subthemes from thematic analyses

*Main themes*	*Subthemes*
Easy and effective	Easy to learn
	Easy to do – no need for equipment
	The effect is immediately observable/visible
	Emphasized as easier than other methods
Experience-based only	Relies on others’ experience
	Seen it, done it and it works
	Insufficient research
	Uncertainties/doubts
First-line response	Facilitates preparation of other treatment
	Overview of the situation
	Used in combination with other methods
Reduces blood loss and improves health	Temporary reduces bleeding
	Less blood loss
	Can save life

### Easy and effective

All of the participants spoke repeatedly about how easy the procedure is to learn, perform and teach. Some emphasized that it is an easy procedure because its effect is immediately visible and tactile. They explained that, during compression, one can feel the pulsation from the aorta with one’s own hand and know that the compression is correctly positioned. One can also instantly see the reduction in bleeding. When explaining how simple the procedure is to learn and teach, one midwife said that it can be easily ‘self-taught’ by reading a textbook and then trying the procedure. Most of the midwives highlighted that a simple video was frequently used as an instructional tool, requiring only a brief training session. One midwife elaborated:

*‘One advantage is, I think, that it's very easy to use. It was very simple to train . . . For example, if you don't have any pulsation in the groin, then you know that the implementation is correct. It's this simple. Everyone can be good at it . . . Everyone understands immediately. So, I think it's very easy to remember. And then you see the result immediately: The bleeding stops and there is no pulsation in the groin.’* (Participant 7)

One midwife stated that she often trained patients’ family members in the procedure, in cases where the patient needed to be transferred far away for further treatment. The midwives described the procedure as easy since only one’s hands are needed:

*‘But just by using this aortic compression it worked and it worked very well. It is fascinating to see how it suddenly stops bleeding. It is a very simple measure. You don’t need any equipment.’* (Participant 2)

In the interviews, the midwives sometimes compared aortic compression with other procedures, such as the use of bimanual uterus compression. They noted that other procedures are harder to perform and require more knowledge about the uterus. They pointed out that, in specific instances, it is easier to perform an aortic compression rather than other procedures, such as during transport, while suturing a tear in the vaginal tract, or while diagnosing the cause or location of the bleeding. Some midwives also emphasized that the different procedures complement each other and may have different desired effects:

*‘We used aortic compression, and it was efficient. Personally, I think it's much easier to find the aorta and give compression then to try to find the fundus and apply pressure. Sometimes the fundus is so loose and difficult to find, and the bleeding is severe . . . I have had deliveries in the ambulances. And this is important, instead of bimanual compression . . . Bimanual compression is almost impossible to do alone, but aortic compression you can do during transport and alone.’* (Participant 4)

### Experience-based only

Most of the participants said that they lacked previous knowledge about aortic compression until someone else introduced it to them. Only one participant thought she had heard about it during her midwifery training, but she did not use it until she began doing humanitarian aid work in places where hospital resources were scarce. Another said that the first time she used aortic compression was when her colleague asked her to do it. The midwives stated that the procedure usually began being used on the ward once someone recommended doing it. One midwife recalled:

*‘You know, I can't quite remember the reason for me using it the first time. Actually, it might have been that the doctor said, “Mariel, can you perform an aortic compression?”. She wanted me to do it while she prepared another treatment . . . So, after having done it once and seen that it worked, it's easier to use it again.’* (Participant 6)

As this quote illustrates, after performing the procedure once and seeing its rapid effect, the midwives continued using it. One midwife said that she continued performing the procedure in hospitals where it was not part of the standard protocol. She explained that it is the procedure she feels most confident with, and she believes it is the best method. Another midwife explained that she introduced aortic compression in her hospital after she returned from humanitarian aid work, where she had performed it.

One of the midwives reported a positive experience with aortic compression, but she still wondered, ‘Could I be wrong?’. This uncertainty stemmed from a lack of sufficient scientific evidence for the procedure – though she was certain that it reduces blood loss. Other participants also expressed the desire for more research to ensure that the procedure is safe and does not interfere with other treatments. As one midwife explained:

*‘But maybe if the compression is sustained over time, there might be some consequences. I don't know. Maybe in coherence with medication given. Or that it affects the blood circulation, if the compression lasts for a longer time period. I don't know.’* (Participant 3)

Despite some uncertainty, it seems that the midwives continued using aortic compression due to their own personal, positive experience with the procedure during PPH.

### First-line response

Aortic compression was seen as a first-line response. As one midwife described it, as soon as a hemorrhage was suspected, aortic compression should be the first measure. Two main reasons were given for its rapid use. First, the compression would prevent the PPH from getting out of control and thus give time for other treatments to be initiated:

*‘Actually, if you don't have control over the bleeding and time is ticking . . . And it runs quickly – the bleeding is quick. And then you have a few minutes for the oxytocin drip to flush. So, then we would use it immediately. Actually, it's effective. It truly is.’* (Participant 1)

Second, the midwives explained that aortic compression might provide a better overview of the situation. PPH is an emergency situation, and many procedures are required simultaneously. Therefore, this ‘pause’ in the bleeding process gives healthcare workers the time they need to diagnose, initiate treatment, transfer to the operating room or stabilize the woman. Aortic compression is further described to improve the visual overview during suturing, owing to the reduced blood in that area. One of the participants described using compression as a tool during diagnosing, when there is uncertainty regarding where the bleeding originates. And several of the participants mentioned situations where aortic compression was important for keeping a woman’s condition stable.

Aortic compression was described as a first aid measure – as a procedure to reduce bleeding. However, it was not regarded as a treatment for the cause of the hemorrhage, nor considered enough on its own. Midwives viewed it something to be used alongside common PPH treatment procedures. As one articulated:

*‘[Aortic compression] won't stop the cause of the bleeding. The cause needs to be identified and eliminated if the compression has an effect. But we use it to reduce the blood loss, to have healthier women and to reduce blood transfusions.’* (Participant 3)

### Reduces blood loss and improves health

The midwives considered the procedure’s main benefit to be the visible reduction in blood loss; in many instances, they described an almost complete cessation of the bleeding, which in some cases felt critical:

*‘[We] ran into the operating room and the doctor was eager to start with procedures and to see the [amount of] bleeding. So, she asked me to decrease the compression so that she could see it. But then I was told, “Don't let go! Don't release!”. Because the bleeding was too severe.’* (Participant 2)

The midwives repeatedly commented on how the bleeding decreased with aortic compression, and that it can therefore be used in different situations: for example, during patient transfer to an operating room or hospital, while perineal tears are sutured or when a reduction in bleeding is needed to allow other treatments to be implemented or take effect. Compression was thus considered vital:

*‘Put simply, [we use it] to minimize and hence to reduce the bleeding. In our hospital we hold the aortic compression all the way into the operating room, until they have sedated that patient . . . We are in the [patient's] bed, while the patient is being transferred, we continue to compress to reduce the blood loss.’* (Participant 3)

Most notably, the midwives described episodes where they believed that aortic compression had saved lives, as severe bleeding can be life threatening:

*‘[The] advantage, I would say, is to buy time to save the [woman's] life, simply said. There's a limit to how much you can bleed. And if the bleeding is severe, this limit is reached quickly. And then [it's crucial] to reduce the large amount of blood loss, temporarily, while you have a chance to stabilize with fluid, to gain control over the situation.’* (Participant 5)

## DISCUSSION

Findings show that the midwives experienced aortic compression to be an efficient, effective method to implement, and the procedure easy to learn, perform and teach. They considered aortic compression a first-line response and were confident that it reduced blood loss. However, most participants had little previous knowledge about aortic compression until someone introduced it to them; as a result, their use and evaluation of the procedure was based on their experience, rather than scientific evidence. The quote ‘Seen it, done it and it works’ – captures the participants’ experience with aortic compression.

### Easy and effective

‘Easy’ and ‘effective’ were the most common adjectives used to describe the advantages of aortic compression. These advantages were linked to teaching and performing the procedure, and its immediate and visible effect. The study’s main themes captured these advantages, and they are discussed below.

### Experience-based only

Though aortic compression is mentioned in national and international guidelines^4,18^, the procedure is under-recognized and poorly researched, nor part of most hospitals’ protocols in Norway. Most of the interviewed midwives had learned the method via YouTube videos or textbooks, or by relying on knowledge shared by their colleagues^11,19^, rather than through formal educational institutions. Routine use was thus often rooted in personal experience.

Evidence-based clinical practice is highly regarded within medical care^20^. Evidence is considered a combination of the individual health worker’s expertise and the best available research^21^. Research regarding aortic compression, however, is scarce. A peer-reviewed article from 2020 identified only 16 case articles from 1946–2019^22^. And while the WHO recommends aortic compression, the recommendation is categorized as ‘weak’ due to lack of research^4^. There are some scattered case articles that describe aortic compression as ‘lifesaving’, but these are not always in relation to PPH^23-25^.

This lack of research might be the procedure’s largest pitfall: As we found in our study, though practitioners may find the procedure effective, they require evidence to validate their personal experience. Nevertheless, it should be noted that the use of experience-based knowledge reflects an ability to integrate one’s own personal knowledge with the patient’s best interest^21^.

### First-line response

The treatment protocol for PPH is comprehensive, and prompt treatment requires several healthcare workers^4^. Causal and supportive treatment is time consuming, and not always sufficient^10^. The midwives in this study used aortic compression prior to and alongside other methods. They were well aware that the method only reduced blood flow and did not treat the cause of the bleeding. During the interviews, the midwives described several episodes where aortic compression ensured the patient’s stability or gave a proper overview of the situation. The midwives thus found aortic compression especially useful during PPH.

In situations where causal treatment is not enough to stop the bleeding, uterine balloon tamponades, anti-shock garments, REBOA or embolization may be used^7-9,26^, all of which take time and resources. In contrast, the midwives frequently highlighted the rapid effect of aortic compression, and that it required only one’s hands. As aortic compression was also used by the midwives to gain time, the midwives saw it as a first-line response. Its immediate effect ensured time to initiate treatment, suture tears, or enable transfer.

### Reduces blood loss and improves health

PPH is a serious complication associated with childbirth. Without prompt, effective treatment, blood loss can quickly become a major complication and life threatening^10^. Globally, PPH is the main cause of maternal mortality and morbidity^3,27^. Indeed, 28% of mortalities are due to bleeding and 50% of major complications are due to PPH. An increase in PPH has also been identified^28^. Reducing blood loss is therefore essential, prompting the need for treatments that are easy and effective^9,29^.

All the midwives described the method as easy, with rapidly visible results. The fact that aortic compression is easy and effective makes it essential in the work to reduce maternal mortality and morbidity. Some of the midwives had experience from humanitarian aid work and saw the method’s usefulness when resources were scarce.

The midwives made it clear that aortic compression does not treat the bleeding. Nevertheless, participants found aortic compression to be an important tool in reducing blood loss and thereby morbidity and mortality.

### Strengths and limitations

It is important to acknowledge the potential limitations of this study. While we have strived to provide a comprehensive analysis of midwives’ experiences with aortic compression during postpartum hemorrhage within the Norwegian context, we acknowledge the need for further investigation. Only seven midwives participated in this study, limiting its external validity, especially since there are few studies with which to compare findings. However, we sought to achieve both data saturation, signifying the point where no new significant insights were obtained from the interviews, and study saturation, encompassing all aspects of the study from participant recruitment to data collection and analysis. It became evident during the analysis that no new substantial information or themes emerged in the later interviews, affirming the study’s internal validity.

Another limitation may be that the first author, who conducted all the interviews, is a midwife and is convinced that aortic compression is an efficient, effective procedure; while she aimed for an objective approach to the interviews and data analysis, this may affect the validity of the study. However, several steps were taken to minimize bias in the data analysis-stage; the two other authors participating in the data analysis and interpretation were unfamiliar with the procedure. Additionally, we followed a rigorous qualitative research method to minimize the potential for researcher bias, such as using open-ended questions and conducting thematic analysis.

Participant bias is another possible limitation when using a convenience sample. However, it can be considered a strength that the midwives came from different areas of Norway and had varied experiences. The situations in which they had used aortic compression varied, from highly technical healthcare systems to more aid-based healthcare systems, but the participants reached the same conclusions regarding the procedure’s advantages.

## CONCLUSIONS

The experiences of seven midwives are not enough to change common PPH treatment strategies. However, the midwives’ positive experiences with the efficiency and effectiveness of aortic compression warrant further investigation of this procedure. PPH remains a leading cause of maternal morbidity and mortality, and more rigorous studies with larger populations and objective data outcomes are necessary. Studies are needed to assess its effect on the amount of bleeding, in particular investigating endpoints such as hemoglobin measurements with and without this procedure. Routine registration of the procedure having been used over an extended period of time, the length of time it was used, and other methods that were implemented, would enable the procedure’s long-term effect to be explored. Future research should also investigate how women experience the procedure.

## Data Availability

The data supporting this research cannot be made available for privacy or other reasons. Approval from the Norwegian Centre for Research Data required data to be deleted upon completion of the analysis.
