# Supplementary material for: Midwives’ experiences with aortic compression for postpartum hemorrhage: A qualitative study
Source: Eur J Midwifery. 2023 Nov 23;7:35. doi: 10.18332/ejm/172880 (PMC10665914; doi:10.18332/ejm/172880)
Supplement: Supplementary file 1 [file EJM-7-35-s1.pdf]

Appendix 1

Midwives' experiences with aortic compression for postpartum haemorrhage: A qualitative study

1 Interview guide

1. Can you please share your midwifery experience? Where you have worked, for how long etc.?
2. Can you share an episode where you needed to use aortacompression?
3. What considerations do you take while using aortacompression?
4. What advantages do you see by using aortacompression?
5. What disadvantages do you see by using aortacompression?
6. Have you ever experienced that aortacompression didn`t work? Or had little effect? If so, do you have any thoughts on why?
7. Why do you think aortacompression is a lesser-known method?

Added questions

8. Are there any patients where you think aortacompression will not be suited?
9. Where did you learn the method?

© 2023 Kalsvik S. et al.
